# Relationship between NK Cell Activation and Clinical Response in Rheumatoid Arthritis Treated with Rituximab

**Published:** 2009-06

**Authors:** A. Lurati, M. G. Marrazza, K. A. Re, M. Scarpellini

**Affiliations:** *Rheumatology Unit Fornaroli Hospital Magenta Italy, Via Donatore Sangue 53 Magenta Italy 20013, Italy*

**Keywords:** Rheumatoid arthritis, Rituximab, NK cells variation

## Abstract

**Introduction:**

We investigated the relationship between the anti CD20 therapy and the NK cell phenotype in patients with Rheumatoid Arthritis (RA).

**Methods:**

patients with seropositive RA according to the ACR criteria that was refractory to conventional and anti TNF alpha agents were studied. All patients were treated with Rituximab (1.0 g at days 1 and 15). At baseline and day 30 were collected: absolute counts of B cells (CD19+), total T cells (CD3+), helper (CD3+CD4+), cytotoxic (CD3+CD8+) and NK (CD16+CD56+). As NK activation marker was used CD54bright expression. Disease activity was primarily assessed using the the Clinical Disease Activity Index (CDAI); in addition, we calculated the Disease Activity Score 28-joint assessment (DAS28).

**Results:**

18 patients were enrolled (mean age ± SD 58.6 ± 2.8 years old). After the rituximab course, as expected CD19+ cells were not detectable, the cytotoxic lymphocytes and CD56+CD16+ cells downregulated (283 ± 34 and 85 ± 15 respectively), instead an up regulation of CD56+CD16+CD54bright was observed (187 ± 43). The dynamic of NK cells activation was significantly associated with clinical variables (r=0.811, p<0.001).

**Conclusions:**

our data suggest a role of rituximab therapy in varying NK phenotype in patients with RA and show that NK cells activation correlates with clinical response.

## INTRODUCTION

Rheumatoid arthritis (RA) is a chronic disease that leads to inflammation and joint damage. Although the pathogenesis of RA remains incompletely understood, recruitment of immune cells into the synovial membrane, a shift in the phenotype and function of synovial fibroblasts, hyperplasia of synovial lining cells are accepted as pathological coordinated events in RA. Lymphocytes that accumulate in the synovial sublining tissue are often organized in functionally competent germinal center-like microstructures that sustain the chronic inflammatory process and autoimmune reactions in RA. Current therapies target the inflammatory consequences of autoimmune activation with the use of disease modiflying atirheumatic drugs (DMARDs) as methotrexate and biologic DMARD. Despite the efficacy of anti TNF agents (Etanercept, Infliximab, Adalimumab), almost 30% of patients have either no response or no sustained response. Several lines of evidence suggests the importance of B lymphocytes in RA (1, 2). Rituximab (RTX) is a monoclonal antibody against CD20 used in cases of rheumatoid arthritis that fail to respond to anti TNF agents. The exact mechanism of how RTX influences the immune responses in autoimmune disorders leading to clinical remission is not known yet. Furthermore, there is not a clear association between clinical improvement after RTX therapy and B cell depletion or rheumatoid factor reduction (3). As known in RA, most of the immune cells interact in complex networks that lead to tissue-injurious inflammatory reactions, i.e. new evidence arise regarding the involvement of natural killer (NK) cells in RA.

In the current study, we determined the NK cells (defined as CD56+CD16+ cells) phenotype cells in patients with rheumatoid arthritis (RA) treated with a single course of rituximab. Therefore, we examined the relationship between the CD16 or CD54 expression on NK cells and the clinical response assessed with CDAI and DAS28.

### Patients and methods

We investigated all patients with RA positive for rheumatoid factor that fulfilled the revised criteria for the classification of RA and previously treated with methotrexate alone and at least one anti TNF agent without adequate clinical response. Subjects eligible for enrolment were adult patients who had active disease defined by Disease Activity Score >3.2 (4).

### Flow cytometric analysis

Fresh blood was collected by venipuncture in EDTA coated vials. Immunophenotyping was performed with the following monoclonal antibodies (mAbs) conjugated with fluorescein isothiocyanate (FITC), phycoerythrin (PE), or phycoerythrin cyanine 5 (PE-Cy5): CD3, CD4, CD8, CD19, CD54, CD16, CD56 (Immunotech/Coulter, Marseille, France). Isotype-matched negative control mouse IgG1-FITC, PE or Cy (MOPC-21) mAbs. Fluorescence was measured using a FACScan (Coulter Electronics, Miami, FL). Briefly, 100 μL of blood were labeled with 10 μL monoclonal antibodies for 20 min in dark. Red blood cells were removed by incubating the samples with lysing solution for 10 min in dark. Tubes were centrifuged for 5 min at 1200 rpm. Samples were treated further with washing solution and were centrifuged 5 min at 1200 rpm. Finally, cells were suspended in cell fixation solution and were ready for flow cytometry measurement. Lymphocytes were gated based on their forward and side scatter characteristics. Informations regarding the percentages of peripheral T (CD3+), T helper (Th: CD3+CD4+), T cytotoxic/suppressor (Tc/s: CD3+CD8+) lymphocytes, B lymphocytes (CD19+) and NK cells CD (CD16 + CD56+) expression were obtained. As reported by Bowles J. *et al*. (16), quantification of the number of NK cells with bright expression of CD54 is a reproducible marker for NK activation induced by mAb-coated tumor cells. We therefore evaluated the number of CD54bright NK cells as a measure of NK-cell activation.

### Disease activity

Disease activity was assessed using the following composite indices: the Clinical Disease Activity Index (CDAI) and the Disease Activity Score with 28-joint assessment (DAS28) Calculations of the DAS28 and SDAI were based on the following: numbers of swollen and tender joints (swollen joint count [SJC] and tender joint count [TJC]), employing the 28 joint count; evaluator’s and/or patient’s global assessment of disease activity (EGA, PGA); and CRP or ESR. The following formulae are the basis for their calculation:

DAS28 = (0.56 × TJC1/2) + (0.28 × SJC1/2) + (0.7 × ln [ESR]) + (0.014 × PGA [in mm])

CDAI = SJC + TJC + PGA (visual–analogue scale [VAS; in cm]) + EGA (VAS [in cm])

## RESULTS

A group of 18 outpatients with RA, 15 females and 3 males, was enrolled. As expected, CD19+ cells depletion occurred in all 18 patients 4 weeks after first infusion (baseline count 268.9±62 vs 1 month 13.1 ± 4.9, p<0.001). No significant variation in CD3+CD4+ cells was detected (1030.8 ± 150.2 vs 1071.3 ± 156.1, p>0.1).

**Figure 1:**
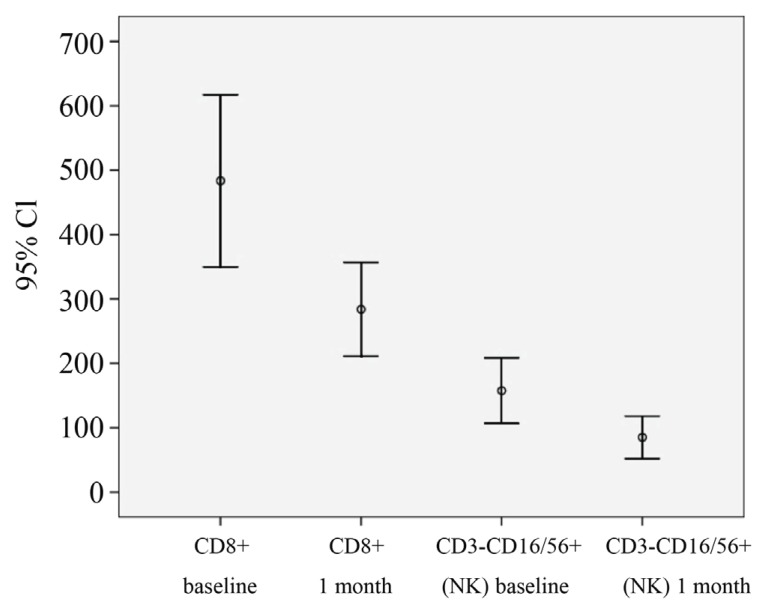
The frequency of CD3+CD8+ and CD56+CD16+ cells.

**Figure 2:**
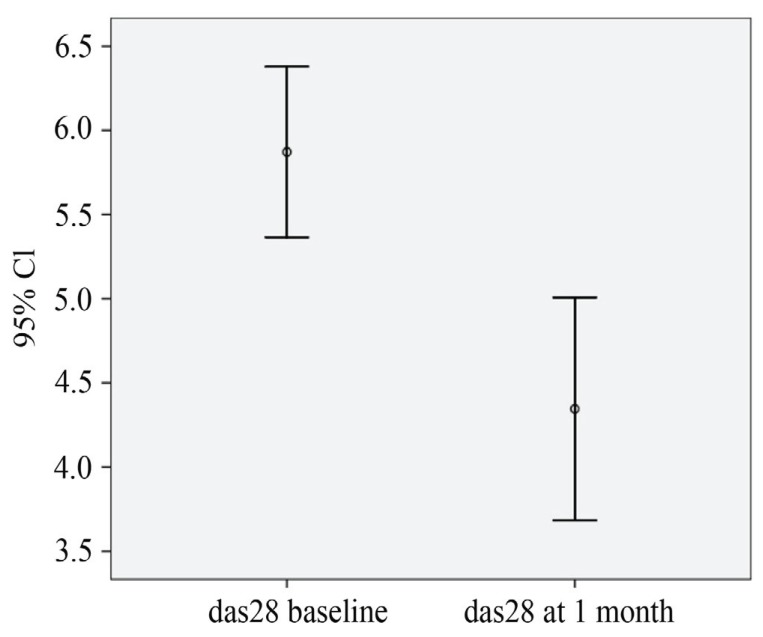
The DAS28 improvement after rituximab treatment.

**Figure 3:**
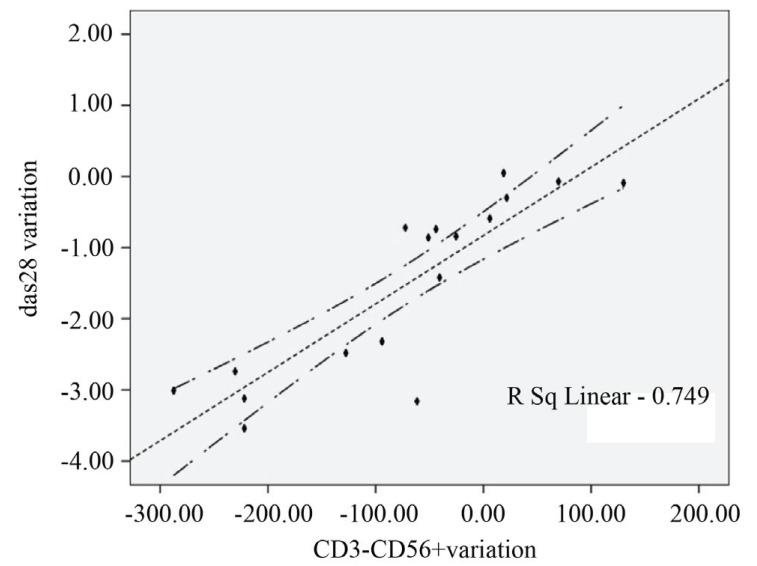
Correlation of EULAR and CDAI response rate with NK cells phenotype modification.

The frequency of CD3+CD8+ (483.5 ± 63.2 vs 283.34 ± 32.3 p<0.01) and CD56+CD16+ cells (157.4 ± 23.5 vs 85.15 ± 2.7) were significantly lower than at baseline (Figure 1), instead an up regulation of CD56+ CD16+ CD54brigth cells was present (34.2 ± 2.2 vs 187 ± 43; p<0.01) The dynamic of NK cells activation was significantly associated with clinical variables (r=0.811, p<0.001). After rituximab treatment was started, the DAS28 improved significantly, resulting in a mean (± SEM) value that was significantly better than the value just before starting therapy (5.8 ± 0.9 vs 4.36 ± 1.2, p<0.01) with a mean difference of -1.5 ± 1.2 (Figure 2). The clinical response in terms of CDAI was similar (58.8 ± 18 vs 37.8 ± 22, p<0.01; mean difference 15 ± 11). The EULAR and CDAI response rate shown a statistic correlation only with NK cells phenotype modification (standard r Pearson index 0.7) (Figure 3).

## DISCUSSION

The role of B cells in immunopathogenesis of RA has not been fully characterised but several possible mechanisms of action have been proposed; B cells may function as antigen presenting cells with co-stimulatory signals required for T cells CD4 regulation, as well they may secrete proinflammatory cytokines (TNF, IL6, other chemokines) and regulate immune response during RA contributing to the inflammation and bone erosions. Recent evidences have arised regarding the involvement of natural killer (NK) cells in RA. NK cells originate from CD34+ hematopoietic progenitor cells and have been defined by flow cytometry as CD56+/CD16+ typical adult or mature cells, CD56+/CD16– immunoregulatory cells, and CD56–/CD16+ cytotoxic cells (5-7). NK cells were detected in the synovium and elevated concentrations were recorded in peripheral blood. NK might contribute to the pathogenesis of RA by perforin- or granzyme-mediated cytotoxicity and cytokine production; Interleukin-15 (IL-15) stimulates the expansion and activation of NK cells. Increased levels of IL-15 in the serum and inflamed synovial joints are characteristic among RA patients. Finally D’Orazio and Stein Streiler showed that activated NK cells can function as super antigen presenting cells that induce non specific stimulation of T lymphocytes (8-12). The CD20 specific antibody Rituximab has been proven to be a successful treatment for B cell malignancies and recently for RA. The binding of rituximab to CD20+ B cells results in B cell depletion through 3 mechanisms of action: antibody dependent citotoxicity, antibody dependent cell mediated cytotoxicity, CD20 B cells apoptosis. The depletion of peripheral B cells occurs immediately following the 2 infusions of rituximab but different levels of clinical response have achieved and therefore it is not possible to predict response on the basis of initial peripheral B cell depletion (13-15). Natural killer cells appear to play a central role in mediating the effects of monoclonal antibody therapy including rituximab as effector cells of antibody-dependent cellular cytotoxicity (ADCC) We evaluated the relationship between natural killer (NK)-cell activation (defined as an up regulation of CD54) mediated by rituximab, NK cell-mediated ADCC and clinical response. Our chief finding is that the activation of NK cells mediated by rituximab, evaluated as up regulation of peripheral CD54 bright NK cells concentration, it’s significantly related to early DAS28 and CDAI responses. Further studies are required to clarify the relationship between B cell depletion, immune network and clinical response in patients with RA.
